# Clinical Outcomes of Soft Tissue Sarcoma around the Elbow Joint: A Retrospective Single Institution Study

**DOI:** 10.1155/2022/1087726

**Published:** 2022-12-17

**Authors:** Edelyn S. Azurin, Norio Yamamoto, Katsuhiro Hayashi, Akihiko Takeuchi, Kaoru Tada, Shinji Miwa, Kentaro Igarashi, Takashi Higuchi, Hirotaka Yonezawa, Sei Morinaga, Yohei Asano, Shiro Saito, Hiroyuki Tsuchiya

**Affiliations:** ^1^Department of Orthopedic Surgery, Kanazawa University Graduate School of Medical Sciences, Kanazawa, Japan; ^2^Department of Orthopedics, Jose B. Lingad Memorial General Hospital, San Fernando, Pampanga, Philippines

## Abstract

**Background:**

We report a retrospective case series analysis of clinical outcomes of patients with soft tissue sarcoma around the elbow.

**Methods:**

Twenty-two patients underwent surgical tumor excision between January 1999 and May 2017, with a mean follow-up of 85.2 months.

**Results:**

Six tumors were localized in the upper arm, nine in the elbow, and seven in the forearm. Sixteen tumors were deep-seated, and six were superficially located. Fifteen patients underwent wide excision, including one amputation, and 18 achieved (81.8%) negative margins histologically. Two local recurrences and four distant metastases developed. The mean Musculoskeletal Tumor Society score was 92.0% (range, 33.3–100). The 5-year local recurrence-free survival rate, metastasis-free survival rate, and overall survival rate were 90.0%, 77.0%, and 79.7%, respectively.

**Conclusions:**

Local control and limb function can have favorable outcomes when the tumor excised has a histologically negative margin without sacrificing the major structure.

## 1. Introduction

Soft tissue sarcomas (STS) are rare heterogeneous tumors that account for <1% of all malignancies [1]. In general, 60% of STS in adults occur in the extremities [2], and only 15% occur in the upper limbs [2–4]. STS account for approximately 0.5% of all cancer-related deaths each year [3]. Approximately, 50% of STS of the upper extremities arise in the shoulder-upper arm region, 30%–40% in the elbow-forearm region, and only 10%–20% in the wrist-hand region [5]. The incidence of STS around the elbow is reported to be 3.8% of all soft tissue tumors [6]. At present, surgery with wide margins is still the treatment of choice for localized STS in the extremities [7], and limb salvage surgery is preferred to maintain upper extremity function [3]. The elbow is a hinge joint that connects the upper arm to the forearm. In the elbow, many anatomical structures allow significant movement for flexion and extension function, facilitate supination and pronation of the forearm, and enable wrist and hand movements. Thus, the resection of a tumor around the elbow is a major challenge that requires careful preoperative surgical planning because of its complexity. Achieving adequate surgical margins is difficult due to the presence of neurovascular structures, lack of expandable soft tissue, and loss of function after surgery [1, 3, 8, 9]. Nonetheless, the primary goal of treatment is to prevent local recurrence and preserve the limb function.

In this study, we aimed to report clinicopathologic data of patients with STS around the elbow joint, treatment, and outcomes. Treatment success was evaluated with a specific focus on patient oncological and functional outcomes.

## 2. Materials and Methods

This descriptive, retrospective study involved patients with STS located around the elbow who were treated and underwent surgical excision of the tumor in our institution between January 1999 and May 2017. Demographic characteristics (age and sex), tumor-specific factors (site, histology, size, depth, surgical stage, and histologic grade), and treatment-specific factors (surgical margin and histological margin) data were collected from the patient medical records and reviewed. The definition of wide and marginal margin was according to Enneking's classification [10]: wide means the tumor was resected with the surrounding natural tissue, and marginal means extracapsular resection of the tumor. The microscopical tumor status was graded according to the residual tumor (R) classification: R2 (grossly positive), R1 (microscopically positive), and R0 (microscopically negative) [11]. We divided the tumor location into three groups: the distal part of the upper arm (DUA), the elbow joint (E), and the proximal part of the forearm (PFA). The tumor of DUA and PFA did not overlay the elbow joint. All patients were followed up every 3 months for 2 years, every 6 months until 5 years, and yearly thereafter. If local recurrence or metastases were observed, patients underwent appropriate treatment. The Musculoskeletal Tumor Society (MSTS) scoring system for the upper extremity [12] was used to determine the functional outcome of patients. The scoring system is based on the analysis of six categories (pain, functional activities, emotional acceptance, positioning of the hand, manual dexterity, and lifting ability). Values of 0–5 are assigned based on the established criteria for the six categories. The total score is calculated from the sum of each category and converted to a percentage value. Scoring was performed 2 years after surgery for each patient. Oncologic outcomes were determined at the final follow-up of the patient. Oncologic outcomes are categorized as died of the disease (DOD), died of another cause (DOA), continuously disease free (CDF) and no evidence of the disease (NED). Continuous disease free is described as being disease-free after the treatment given until the last follow-up, while no evidence of disease is described as having a relapse during follow-up and was treated accordingly, and upon the last follow-up, the patient has no evidence of recurrence or metastasis [13]. This study was approved by the ethics committee of Kanazawa University Hospital (no. 2984) and conducted in accordance with the Declaration of Helsinki. Informed consent was obtained from the patients using the opt-out method.

### 2.1. Statistical Analyses

Categorical data were recorded based on the frequency of the events. Continuous variables, such as age, tumor size, and follow-up period were reported as mean or median with standard deviation. The rates of overall survival, local recurrence-free survival, and metastasis-free survival were calculated using the Kaplan–Meier method with the Statistical Package for Social Sciences (SPSS) for Windows (version 25, IBM Corp., Armonk, NY, USA).

## 3. Results

**Figure 1:**
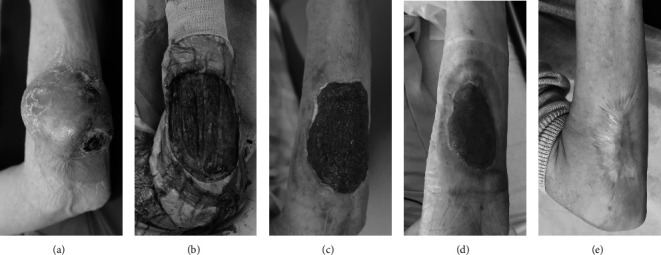
A 67-year-old female with an UPS on the right proximal forearm (case no. 16) (a). The cutaneous defect is 7 × 6 cm after tumor excision (b). The defect is covered with the granulation tissue after 4 weeks (c). The size of skin defect has reduced with the epithelialization after 8 weeks (d). The wound is completely healed after 6 months and shows full range of motion after 20 months (e).

Five hundred sixty-four STS were treated between January 1999 and May 2017 at our institution. Twenty-two (3.9%) tumors were located around the elbow joint and were treated with a mean follow-up of 85.2 months (range, 2.0–180.0). The mean age was 45.9 years (range, 14–78 years). There were eight men (36.4%) and 14 women (63.6%). Six tumors (27.3%) were localized in the DUA, nine (40.9%) in E, and seven (31.8%) in PFA. All cases of E were located over the elbow joint. There were 16 (72.7%) deep-seated tumors and six (27.3%) superficial tumors. The average tumor size based on the greatest diameter was 5.7 cm; deep-seated tumors were larger than subcutaneous tumors (mean size of 6.1 cm and 4.8 cm, respectively). The most common types confirmed histologically were undifferentiated pleomorphic sarcoma (UPS) (*n* = 3, 13.6%) and clear cell sarcoma (CCS) (*n* = 3, 13.6%). Regarding histologic grading, 86.4% (*n* = 19) of the patients had high-grade tumors, and based on surgical staging using the American Joint Committee on Cancer, 50.0% (*n* = 11) was graded as stage IIIA. The surgical procedures were wide excision in 15 patients (68.2%) including one amputation, and two cases were classified as microscopically positive (R1). Marginal excision was performed in six patients (27.3%), and one patient was classified as R1. Intralesional excision (R2) was performed in one patient (4.5%). Eventually, 18 of 22 patients (81.8%) were evaluated as R0. Skin defects occurred in six cases and was reconstructed with a latissimus dorsi flap in one case (Case No. 6) and occlusive dressing in five cases (Cases No. 9, 10, 11, 14, and 16) (Figure 1) [14]. Skin defects were healed in four cases: Cases No. 9, 10, 14, and 16 at 23, 18, 14, and 19 weeks, respectively. One wound (Case No. 11) was not healed until the patient death.

**Figure 2:**
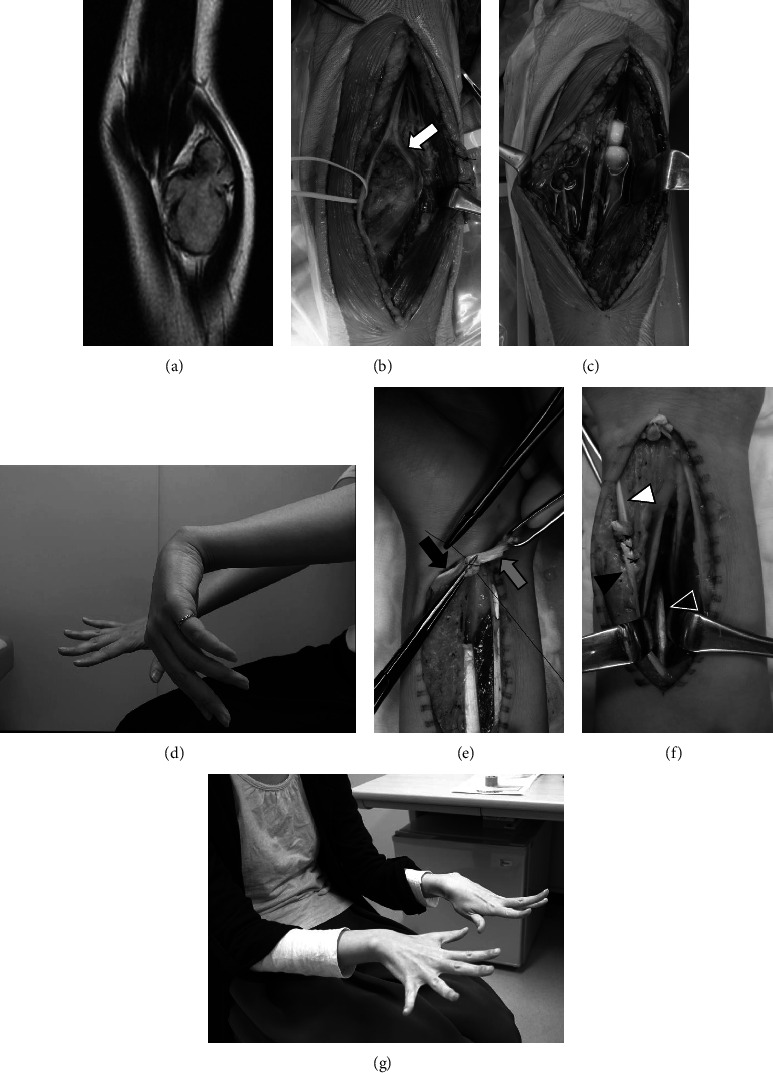
A 23-year-old female with a synovial sarcoma on the left elbow joint (case no. 8). MRI shows the iso-intensity tumor (53 × 32 mm) on T2WI (a). The posterior intraosseous nerve is involved in the tumor (white arrow) (b). The wide excision is performed and combined excision with joint capsule and posterior intraosseous nerve (PIN) (c). The patient shows the PIN palsy after 2 years (d). The tendon transfer (tsuge method) is performed 2 years after the initial surgery. The flexor carpi radialis (FCR) (grey arrow) is transferred to the abductor pollicis longus (black arrow) (e). The palmaris longus (black arrowhead) is transferred to the extensor pollicis longus (white arrowhead) and the FCR (white line arrowhead) is transferred to the extensor digitorum communis (f). The patient demonstrates full extension of fingers 2 years after the second surgery (g).

One case needed a combined resection of the radius and flexor pollicis longus and was reconstructed with a tumor bearing frozen autograft [15] and tendon transfer using palmaris longus (Case No. 15). Palmaris longus transfer was performed at the time of tumor excision in another case (Case No. 12). Eleven patients (50.0%) underwent neoadjuvant chemotherapy, and seven of eleven patients underwent postoperative chemotherapy. One case was administered postoperative chemotherapy due to the confirmation of exact diagnosis as UPS. Only three patients (13.6%) underwent radiotherapy postoperatively because one had local recurrence and two had positive histologic margins. Major nerves were involved in the tumors of three cases (the ulnar nerve in Case No. 6 and 13 and posterior intraosseous nerve in Case No. 8) and were sacrificed. Tendon transfer (Tsuge method) [16] was performed 2 years after the initial surgery (Case No. 8) (Figure 2).

At the time of the final follow-up, 68% (*n* = 15) were CDF and 4.5% (*n* = 1) had NED. Of the 22 patients, 22.7% (*n* = 5) died due to the disease and 4.5% (*n* = 1) died due to another cause (Table 1).

**Figure 3:**
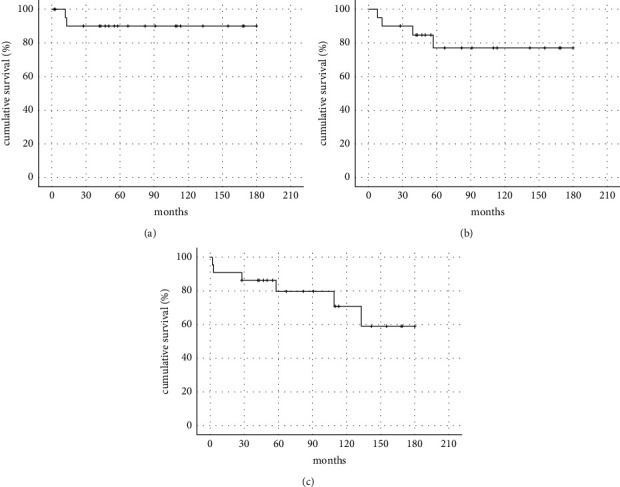
The Kaplan-Meier survival curve of recurrence-free (a), metastasis-free (b), and overall survival (c). The estimated 5-year survival rate is 90.0% (a). 77.0% (b) and 79.7% (c), respectively.

The estimated 5-year local recurrence-free survival rate was 90.0% (Figure 3(a)). Local recurrence occurred in two patients (12 months and 13 months). The estimated 5-yearmetastasis-free survival rate was 77.0% (Figure 3(b)). Metastases developed in four patients, of whom three had pulmonary dissemination and one had axial lymph node dissemination (Table 1) with a mean period of 29 months (range, 8–57). The mean time to death after the development of distant metastasis was 34.2 months (median time to death, 15.9 months). The estimated 5-year overall survival rate was 79.7% (Figure 3(c)).

Functional outcomes were available for 18 patients (81.8%) because two succumbed to the disease shortly after surgery and two patients were not assessed. The MSTS scoring system for the upper extremity revealed a mean score of 92.0% (range, 33.3–100), with most patients being satisfied after the surgery (Table 1). Predictive factors were not analyzed as the number of events was too small to yield statistically significant differences.

## 4. Discussion

In this study, we identified the clinicopathologic data and outcomes of 22 patients with STS around the elbow. Fifteen patients (68.2%) underwent wide excision and 18 (81.8%) achieved negative margins histologically, with an estimated 5-year local recurrence free survival of 90.0% and a favorable mean MSTS score of 92.0%.

Alektiar et al. [8] reported the clinical outcomes of STS arising in the knee and elbow. Their study included 21 patients with elbow STS, wherein local recurrence developed in seven patients (33.3%). Although they analyzed prognostic factors, including knee STS, tumor size (>5 cm) was an independent prognostic factor of local recurrence. Emori et al. [17] reported a large series of STS in the elbow joint. Their study included 219 cases and local recurrence was observed in 21 cases. Their analysis revealed that tumor size (>10 cm) was an independent risk factor of local recurrence. In this study, recurrence was observed in two patients, one with myxofibrosarcoma (Case No. 14) and the other, UPS (Case No. 22). The tumor size was <5 cm in both cases; however, preoperative MRI showed a tail-like pattern in both cases. Surgery was attempted with a wide margin, and one case resulted in a positive margin. Although radiotherapy was performed in both cases, local recurrence occurred. Therefore, an infiltrative-type tumor with a tail-like pattern may be considered to have a risk of recurrence and should be excised with at least a 1 cm wide margin for precise evaluation of tumor extension [18, 19]. Other studies have shown that the strongest predictor of local recurrence is a positive surgical margin, which indicates the presence of residual disease [2, 20]. Additional radiotherapy for patients at high risk of local recurrence, including those with positive or close margins, is usually considered; however, its exact role remains undetermined [8, 9], and attention needs to be paid to the complications of joint stiffness in this area [8].

Gustafson and Arner [9] reported the clinical outcomes of STS arising in the upper extremity. Their study included 50 patients with STS in the upper arm, eight with STS in the elbow, and 40 with STS in the forearm. They reported that local recurrence developed in 15 of 28 patients who received inadequate treatment (surgery with an intralesional margin with or without adjuvant radiotherapy or surgery with a narrow margin without adjuvant radiotherapy). Additionally, local recurrence developed in 16 of 74 patients who received adequate treatment (surgery with a narrow margin with adjuvant radiotherapy or surgery with a wide or radical margin with or without adjuvant radiotherapy). The authors considered that the tumor size in their series was relatively small compared with that in the lower extremity or trunk wall, which resulted in a favorable 5-yearmetastasis-free survival rate (72%).

Regarding a large cutaneous defect, a muscular flap or skin graft are useful options [21]. We treated four skin defects by occlusive dressing that completely healed without additional surgery. Although it took a long time to achieve healing, the occlusive dressing had several advantages such as avoiding sacrificing normal tissue and immediately starting the range of motion training of the elbow joint [14]. We also performed staged tendon transfer for the functional reconstruction of the hand drop due to sacrificing of the radial nerve in Case No. 8. The timing of the reconstruction surgery is still debatable [21–23]. We considered that staged reconstruction surgery is acceptable for functional reconstruction including tendon transfer to determine the risk of local recurrence.

In our series, the majority of histotypes identified were UPS and CCS, which is similar to the findings of other studies [1, 9], wherein UPS was the most common STS in the upper extremity. UPS, previously known as malignant fibrous histiocytoma, often presents in the sixth to eighth decade of life and has a local recurrence rate of 19%, a metastatic rate of 35% with the lungs as the most common site, and 5-year survival rate of 65%. Tumor size, depth, and histologic grade seem to correlate with the metastatic rate and ultimate survival rate [3]. In our series, we identified three patients (Cases No. 9, 16, and 22) with UPS in the seventh and eighth decades of life. All patients achieved negative surgical margins, but only one of the three remained disease-free at the last follow-up; this patient had a superficial tumor compared to the other two who had deep-seated tumors. These two patients died on succeeding follow-ups, where one presented with metastasis 8 months after the initial treatment but died of another cause at 58 months (Case No. 9). The other presented with both metastasis and local recurrence at 12 months postoperatively but succumbed to the disease after 28 months (Case No. 22). Even with limited cases, our data are comparable to those of other studies [1, 3, 9, 17].

In contrast, CCS are exceedingly rare tumors and their incidence has been reported to be <1% of all STS cases [24]. CCS is a subtype specific to the hand and wrist [25], common in young adults aged between 20 and 40 years, and is usually deep-seated, with a local recurrence rate of 21%, metastatic rate of 69%, and 5- and 10-year overall survival rates of 47% and 36%, respectively [26]. Even after radical surgery and adjuvant treatment, these tumors commonly metastasize with a propensity to spread to the regional lymph nodes, affect 10%–14% of the patients, and have a poor prognosis [27]. Tumor size is also an important factor, as tumors measuring <5 cm have a better prognosis than those measuring >5 cm [26]. However, in our series, CCS was one of the most commonly identified tumors (13.6%, *n* = 3). Emori et al. also reported the relatively high incidence of CCS in the elbow (7/216, 3.2%) [17] compared to the reported incidence in all STS cases [25]. All patients in our series were within the age range of 20–40 years and received chemotherapy. Only one of the three identified patients had superficial and small tumors (Case No. 3), achieved a negative surgical margin after resection, and was continuously disease-free since the latest follow-up. The other two patients (Case No. 2, 11) had deep-seated tumors measuring >5 cm and initially presented with metastases. One patient underwent amputation but still developed another metastasis and died from the disease 3 months later (Case No. 2). The other patient underwent intralesional resection and died from the disease 2 months later (Case No. 11). This justifies the findings of the other studies [3, 26] indicating that CCS has a dismal prognosis once large in size and presenting with metastasis.

Our estimated 5-yearmetastasis-free survival rate was 77.0%, which is higher than the 68% reported by Popov et al. [1], probably because we had fewer patients in our study. STS has a propensity to metastasize to the lungs, with up to 80% of metastatic STS metastasizing to the lungs [5]. Four of our patients developed metastases, of whom three had pulmonary dissemination (Cases No. 6, 15, 22) and one had axial lymph node dissemination (Case No. 8).

Poor prognosticators including a high histologic grade, size >5 cm, deep location, and tumor stage have been identified [3, 5]. Of these, histologic grade has the highest impact on systemic control after surgery [5]. In our series, 19 (83.36%) patients had high-grade tumors; deep-seated tumors were larger than the subcutaneous tumors, with a mean size of 6.1 cm and 4.8 cm, respectively; and the most common stage was grade IIIA. In our study, all patients who died had high-grade, deep-seated tumors, and 80% of the deceased patients had a tumor stage of IIIA or higher. Metastatic relapse after complete surgery occurs in approximately 40% of patients, leading to death from the disease within the first 8 years after the initial diagnosis [5], which is higher than that observed in our study (22.2%). Murray [3] reported that the 5-year overall survival rate is between 75% and 80%, which is comparable to ours at 79.7%. A multidisciplinary approach is necessary for systemic treatment [28].

This study had several limitations. It was a single-center, retrospective review of patients with soft tissue sarcoma around the elbow, having a small sample size and a heterogeneous population. The indications for chemotherapy and radiotherapy varied in each case. The tumor site was divided into three groups, and tumors located in the DUA and PFA did not cross the elbow joint; however, those tumors were close to the elbow joint and were considered to have influenced the postoperative elbow joint function.

## 5. Conclusions

In conclusion, STS around the elbow present a treatment challenge due to their low incidence and atypical anatomic and histopathologic features. Our study, similar to others, also suggests that local control and limb function can have favorable outcomes when the tumor is excised with histologically negative margins without sacrificing the major structure. However, regardless of the treatment, long-term vigilance is required for these patients.

## Figures and Tables

**Table 1:** Patient characteristics, disease features, treatment details, and outcomes of study population.

Case/age/sex	Histology	Site	Size (mm)	Depth	American Joint Committee on Cancer (AJCC) staging				Surgery	Combined resection	Reconstruction	R-classification	Adjuvant treatment	*L*/*R*(mos)	Mets (mos)	Musculoskeletal Tumor Society (MSTS) scoring system							*F*/*U*(mos)	Outcome
					T	N	M	Stage								Pain	Function	Emotional acceptance	Hand position	Dexterity	Lifting ability	Score (%)		
1/m/35	ALT	Elbow	70	D	T2	N0	M0	IB	Marginal			R0				5	5	5	5	5	5	100	66.9	CDF
2/f/43	CCS	Elbow	52	D	T2	N1	M0	IV	Wide (amp)			R0 (amp)	Cx (neo)			N/A	N/A	N/A	N/A	N/A	N/A	N/A	2.7	DOD
3/f/38	CCS	Elbow	27	S	T1	N0	M0	II	Marginal			R0	Cx (neo & adj)			5	5	5	5	5	5	100	91.4	CDF
4/f/36	MLS	Elbow	72	S	T2	N0	M0	IIIA	Marginal			R0	Cx (neo)			5	5	5	5	5	5	100	27.5	CDF
5/f/22	LGFS	Elbow	48	D	T1	N0	M0	IA	Wide			R0				5	5	5	5	5	5	100	42.4	CDF
6/m/70	MPNST	Elbow	75	D	T2	N0	M0	IIIA	Wide	Ulnar N	LD flap	R0	Cx (neo & adj)		Yes (57, lung)	5	2	2	4	3	5	70	133.3	DOD
7/f/36	PNET	Elbow	32	S	T1	N0	M0	II	Wide			R0				5	5	5	5	5	5	100	82.1	CDF
8/f/22	SS	Elbow	50	D	T2	N0	M0	IIIA	Wide	Radial N	TT (2^nd^ surgery)	R0	Cx (neo & adj)		Yes (39, lymph node)	5	5	5	4	4	5	93	109.3	DOD
9/m/76	UPS	Elbow	75	D	T2	N0	M0	IIIA	Wide		OD	R0			Yes (8, lung)	N/A	N/A	N/A	N/A	N/A	N/A	N/A	57.5	DOA
10/f/23	AS	PFA	70	S	T2	N0	M0	IIIA	Wide		OD	R0				5	5	5	5	5	5	100	154.9	CDF
11/m/25	CCS	PFA	78	D	T2	N0	M1	IV	Intraregional		OD	R2	Cx (neo & adj)			N/A	N/A	N/A	N/A	N/A	N/A	N/A	2.0	DOD
12/f/67	LMS	PFA	35	D	T2	N0	M0	IIIA	Wide		TT	R0	Cx (neo & adj)			N/A	N/A	N/A	N/A	N/A	N/A	N/A	54.6	CDF
13/f/71	MGCT	PFA	92	D	T2	N0	M0	IIIA	Marginal	ulnar N		R0				5	4	4	4	4	5	87	180.0	CDF
14/f/67	MFS	PFA	32	S	T1	N0	M0	II	Marginal		OD	R1	Rx	Yes (13)		2	1	1	3	0	3	33	141.7	NED
15/f/14	RS	PFA	20	D	T2	N0	M0	IIIA	Wide	Radius	Frozen autograft & TT	R0	Cx (neo & adj)			5	5	5	4	4	5	93	109.6	CDF
16/f/67	UPS	PFA	55	S	T2	N0	M0	IIIA	Wide		OD	R1	Cx (adj)			5	5	5	5	5	5	100	47.4	CDF
17/m/43	ALT	DUA	134	D	T3	N0	M0	IB	Marginal			R0				5	5	5	5	5	5	100	49.8	CDF
18/m/21	ASPS	DUA	25	D	T1	N0	M0	II	Wide			R0	Cx (neo)			5	5	5	5	5	5	100	168.4	CDF
19/f/58	LMS	DUA	65	D	T2	N0	M0	IIIA	Wide			R0	Cx (neo & adj)			5	5	5	5	5	5	100	113.0	CDF
20/f/76	MFS	DUA	50	D	T2	N0	M0	IIIA	Wide			R0				5	5	5	5	5	5	100	42.8	CDF
21/m/21	SS	DUA	30	D	T1	N0	M0	II	Wide			R1	Cx (neo), Rx			5	5	5	5	5	5	100	169.4	CDF
22/m/78	UPS	DUA	35	D	T1	N0	M0	II	Wide			R0	Rx	Yes (12)	Yes (12, lung)	3	4	4	4	4	5	80	27.9	DOD

Abbreviations: ALT, atypical lipomatous tumor; CCS, clear cell sarcoma; MLS, myxoid liposarcoma; LGFS, low-grade fibromyxoid sarcoma; MPNST, malignant peripheral nerve sheath tumor; PNET, peripheral primitive neuroectodermal tumor; SS, synovial sarcoma; UPS, undifferentiated pleomorphic sarcoma; AS, angiosarcoma; LMS, leiomyosarcoma; MGCT, malignant granular cell tumor; MFS, myxofibrosarcoma; RS, rhabdomyosarcoma; ASPS, alveolar soft part sarcoma; PFA, proximal forearm; DUA, distal upper arm; D, deep; S, superficial; LD, latissimus dorsi; TT, tendon transfer; OD, occlusive dressing; Cx, chemotherapy, neo, neoadjuvant; adj, adjuvant; Rx, radiotherapy; L/R, local recurrence; Mets, metastasis; CDF, continuous disease free; NED, no evidence of disease; DOD, died of the disease; DOA, died of another cause.

## Data Availability

Data supporting this research are available upon requestfrom the corresponding author.
